# Is Local Nitric Oxide Availability Responsible for Myocardial Salvage after Remote Preconditioning?

**DOI:** 10.5935/abc.20160100

**Published:** 2016-08

**Authors:** Esbeidira Aranet Arroyo-Martínez, Alejandra Meaney, Gabriela Gutiérrez-Salmeán, Juan Miguel Rivera-Capello, Vidal González-Coronado, Alejandro Alcocer-Chauvet, Genaro Castillo, Nayelli Nájera, Guillermo Ceballos, Eduardo Meaney

**Affiliations:** 1 Unidade Cardiovascular - Hospital "Primero de Octubre", Cidade do México - México; 2 Faculdade de Ciências da Saúde, Universidade Anáhuac México Norte, Cidade do México - México; 3 Laboratório de Investigação Integral Cardiometabólica. Seção de Estudos de Pós-graduação e Investigação. Escola Superior de Medicina, Instituto Politécnico Nacional, Cidade do México - México

**Keywords:** Nitric Oxide, Ischemia, Ischemic Preconditioning, Myocardial, Reperfusion

## Abstract

**Background:**

Remote ischemic preconditioning (RIPC) represents an attractive therapy for myocardial protection, particularly when ischemic events can be anticipated. Although several hypothetic mechanisms have been proposed, no definite molecular pathways have been elucidated.

**Objective:**

We evaluated the effect of brachial circulation cuff occlusion on myocardial ischemic tolerance, necrosis, and nitric oxide (NO) in patients with ischemic heart disease undergoing elective percutaneous coronary interventions (PCI).

**Methods:**

46 patients were randomly allocated into two groups: control and RIPC before PCI procedures. Electrocardiographic analysis, serum concentrations of troponin I (cTn-I) were measured at baseline and 24 hours after PCI. A blood sample from the atherosclerotic plaque was drawn to determine nitrate and nitrites.

**Results:**

RIPC increased the availability of NO in the stented coronary artery. Control patients presented a small but significant increase in cTn-I, whilst it remained unchanged in preconditioned group. The preconditioning maneuver not only preserved but also enhanced the sum of R waves.

**Conclusions:**

RIPC induced an intracoronary increase of NO levels associated with a decrease in myocardial damage (measured as no increase in cTn-I) with electrocardiographic increases in the sum of R waves, suggesting an improved myocardium after elective PCI.

## Introduction

Ischemic preconditioning is a well-known phenomenon by which short periods of ischemia-reperfusion provide an increased tolerance to subsequent sustained ischemic episodes.^1^ Remote ischemic preconditioning (RIPC), i.e., the increased myocardial tolerance to ischemic insults after short-term ischemia-reperfusion episodes induced in a distant body tissue or organ, has demonstrated a conspicuous capacity against ischemia, stunning, as well as an ability to limit infarct size in both animal models and humans.^2-5^

Although the specific underlying molecular pathways are yet poorly understood, there is a handful of hypothetic mechanisms explaining this rather intriguing phenomenon. Neural [e.g., bradykinin and nitric oxide (NO)], humoral (e.g., adenosine and angiotensin), and systemic protective response (i.e., suppressing inflammation and apoptosis) premises, acting either alone or intertwined, have been proposed as possible mechanistic processes of RIPC.^6^ The pragmatic implementation of techniques of remote preconditioning uncovers a promising therapeutic field for myocardial salvage and protection, particularly when ischemic events can be anticipated, as in percutaneous coronary interventions (PCI) or aorto-coronary bypass graft surgery, among others.

Interestingly, it has been proposed that NO may play a relevant and perhaps decisive role resulting in myocardial protection. However, to our best knowledge, a direct measure of NO in the coronary territory, after periphery conditioning and just before reperfusion, has not been assessed.

The purpose of this study was to evaluate the effect of brachial circulation cuff occlusion on myocardial ischemic tolerance, myocardial necrosis, NO bioavailability at the site of angioplasty and renal function in patients with ischemic heart disease undergoing elective PCI.

## Methods

Based on the study by Hoole,^7^ in which serum cardiac troponin I (cTn) was increased more than 30% in control patients compared with those who underwent a RIPC 24 hours after elective PCI, a sample size of 14 patients was estimated to provide 80% power and 95% of confidence. Anticipating a 20% of losses, and 15% reduction of elevated cTn-I, a group of 46 patients (23 in each of the control and RIPC intervention groups) was assembled.

Study protocol was approved by both Ethics and Research Institutional Committees and it was conducted according to the Declaration of Helsinki, Good Clinical Practices and Mexican Federal Regulations.^8,9^ We included patients of any gender, aged ≥18 years, who signed an informed consent, had documented clinical evidence of ischemic heart disease, accepted PCI therapeutic procedures and had concentrations of serum cTn-I less than three times de 99^th^ percentile value. Exclusion criteria include: patients with acute coronary syndromes or hemodynamic instability, those requiring immediate angioplasty and stenting procedures, pregnant or breastfeeding women, and those patients medicated with drugs having effects as activators of potassium channels, as glibenclamide or nicorandil.

All patients were medicated with aspirin (300 mg) and clopidogrel (600 mg) the day before the PCI. All selected patients concluded the trial.

One hour before the PCI procedure, a 12-lead electrocardiogram (ECG) was obtained. Then the ECG was repeated 24 hours after PCI. Electrocardiographic analysis comprehended the quantitation of the positive or negative ST deviation, new Q waves, and the occurrence of sudden left bundle branch block (LBBB). The summation of R waves (ΣR), in mm, was carried out in precordial leads and in all 12 leads.

Additionally, a peripheral venous blood sample was drawn to measure serum concentrations of troponin-I by means of monoclonal antibody-immunoassay with a Bayer ADVIA 60 hematology analyzer, whose 99^th^ percentile value is 0.04 ng/mL. The variation coefficient of the assay is less than 10%^10^ in our laboratory. Troponin-I was measured at baseline and 24 hours after PCI.

After recruitment, patients were randomly allocated in two groups, one serving as controls and another in which patients underwent ischemic preconditioning. In these latter subjects, 1 hour before the PCI procedure a RIPC maneuver was carried out with patients lying in dorsal decubitus. RIPC was done in the left arm, using three cycles of cuff occlusion, raising the pressure to 200 mm Hg during a period of 5 minutes followed by a 5-minute period of cuff deflation. Afterwards, all patients underwent a standard PCI procedure, including the stenting of all susceptible and accessible coronary lesions. The different interventional cardiologists involved in the study were blinded to the preconditioning maneuver. Before stenting, the tip of the catheter was placed just in the site of the atherosclerotic plaque to be stented, and a blood sample was drawn to measure nitrate and nitrites (NOx), the degradation products of NO, as an indirect measure of disposability of this gas, which in turn is an emblematic marker of endothelial function. NO metabolites concentrations were measured using a commercially available colorimetric enzymatic Kit (Cayman, Chemicals), following manufacturer's instructions. Optical density was determined at 540 nm. Since NOx is excreted through the kidney, the ratio NOx/creatinine was estimated in order to rule out the influence of any renal impairment.

Angina pain experienced during PCI was quantified with a modified scale of the American College of Sports Medicine^11^ in grade 0 (no pain or discomfort at all), grade 1 (light, barely noticeable), grade 2 (moderate, bothersome), grade 3 (severe, very uncomfortable) and grade 4 (most severe pain never experienced).

Periprocedural myonecrosis/myocardial infarction. Myocardial necrosis was considered when serum concentration of cTn was above the 99^th^ percentile of the reference value.^10^ Notwithstanding, the diagnosis of myocardial infarction was based on the updated criteria of the European Society of Cardiology, the American College of Cardiology Foundation, the American Heart Association and the World Heart Federation (ESC/ACCF/AQHA/WHF) document titled Third Universal Definition of Myocardial Infarction.^12^ This international expert consensus document establishes that the preferred biomarker of necrosis is cTn and at least one of the five additional criteria: ischemic symptoms; new significant ST/T wave changes or new LBBB; new pathologic Q waves; imaging demonstration of new loss of viable myocardium (or new regional wall motion abnormality); and intracoronary thrombus (identified by angiography or autopsy). Following the same document, a periprocedural myocardial infarction was considered when cTn elevation exceeded five times the 99^th^ percentile value of reference in patients with normal basaline values.

Patients who experienced some procedural complications, as coronary dissection, longstanding periprocedural arrhythmias or cardiac arrest, were excluded from the analysis. Thus, only cases with complete successful angioplasty and stenting, such as cases in which all accessible and relevant lesions were treated, without complications, with full intraluminal gain, and flow TIMI 3, were considered for the analysis.

Kidney function was estimated measuring serum creatinine and glomerular filtration rate (GFR) by means of the Cockcroft-Gault^13^ creatinine clearance equation, before and after the PCI procedure:

GFR= [(140 - age) x weight (kg)/(72 x serum creatinine (mg/dL)] x (0.85 in women).

Patients were allocated in the five stages of chronic kidney disease (stages I-V), according to their value of GFR (<15, 15-29, 30-59, 60-89 and >90 mL/min). GFR was estimated before the intervention and 24 hours later.

Statistical analysis. All values were expressed as mean ± standard deviation. Normal distribution of data was analyzed using the Kolmogorov-Smirnov test, differences (before vs. after) in continuous variables were evaluated using Student's paired *t* test, whereas unpaired *t* test was used to evaluate intergroup differences and percentage of change (deltas). Differences between categorical data, i.e., frequencies and percentages, were evaluated with z tests. A p value <0.05 was considered as significant. Prism GraphPad(r) (GraphPad, San Diego, CA, USA) software was used for statistical analysis.

## Results

All recruited patients completed the study protocol. Table 1 gathers demographic and clinical baseline data. Both groups were similar (i.e., no statistical difference was found, p = *ns*) in age, gender distribution, occurrence of adiposity, hypertension and diabetes, previous myocardial infarction and functional renal status. There were more patients in the preconditioning group medicated with β-blockers (5 subjects) and statins (2 subjects, no significant differences), whereas more control patients were medicated with insulin and metformin, in spite of the fact that the proportion of diabetes was similar in both groups. Calcium channel blockers and renin-angiotensin axis modulators were prescribed in similar proportion in both groups. Almost undetectable quantities of cTn were found in all patients, and none had, before the intervention, ST abnormalities.

**Table 1:** Demographic and clinical characteristics of patients

**Variable**	**ControlGroup (n=23)**		**PreconditioningGroup (n=23)**	
	**x**	**SD**	**x**	**SD**
**Age (years)**	66.1	11.3	63	6.9
**Body mass index (kg/m^2^)**	26.8	4.2	27.7	4.5
	n	%	n	%
**Male**	15	65	16	69
**Type 2 diabetes mellitus**	16	79	14	60.8
**High blood pressure**	19	83	21	91.3
**Pharmacological treatment**				
Aspirin	23	100	23	100
Clopidogrel	23	100	23	100
β-blockers	13	57	18	78
ACEI	8	35	13	56
ARBs	11	48	7	30.4
CCB	5	21.7	6	26
Statins	19	82.6	21	91.3
Insulin	5	22	4	17.3
Metformin	16	70	11	47.8
**Previous myocardial infarction**	11	48	12	52
**ST elevation (0.5-1 mV)**	0	0	0	0
**Baseline troponin I <0.04 ng/mL**	23	100	23	100
**Glomerular filtration rate (mL/min/1.73 m^2^)**				
< 15 (renal failure, stage V)	3	13	0	0
15-29 (severe reduction of GFR, stage IV)	4	17.3	3	13
30-59 (moderate reduction of GFR, stage III)	3	13	4	17.3
60-89 (mild reduction of GFR, stage II)	7	30.4	10	43
≥ 90 (normal GFR, stage I)	6	26	6	26

SD: standard deviation; GFR: glomerular filtration rate; ACEI: angiotensin-converting-enzyme inhibitors; ARBs: angiotensin II receptor blockers; CCB: calcium-channels blockers.

Angina pain during or after PCI. Only one patient in each group experienced angina pain (grade 4 in both cases) during or immediately after the angioplasty procedure.

**Figure 1:**
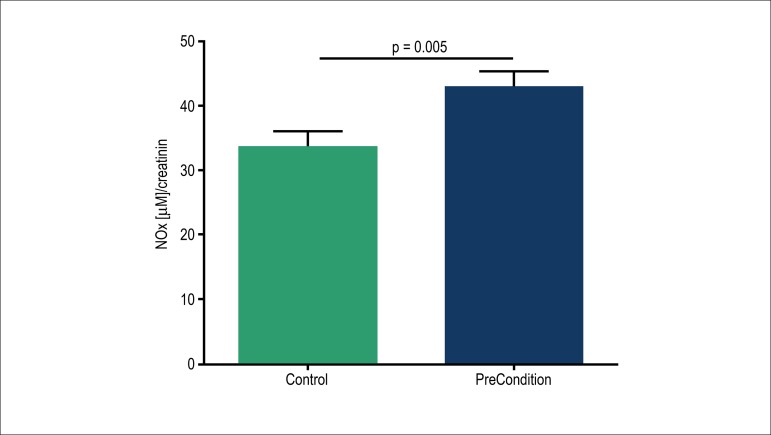
Local nitric oxide (NOx) corrected by creatinine levels in controls and preconditioned patients. Data are expressed as means ± SEM.

NO bioavailability. Figure 1 shows that the concentration of NOx in blood withdrawn from the coronary artery to be stented, just in the proximity of the atherosclerotic lesion, was significantly greater in preconditioned patients. This result points out that preconditioning maneuver increased the availability of NO in the coronary artery selected for stenting.

**Figure 2:**
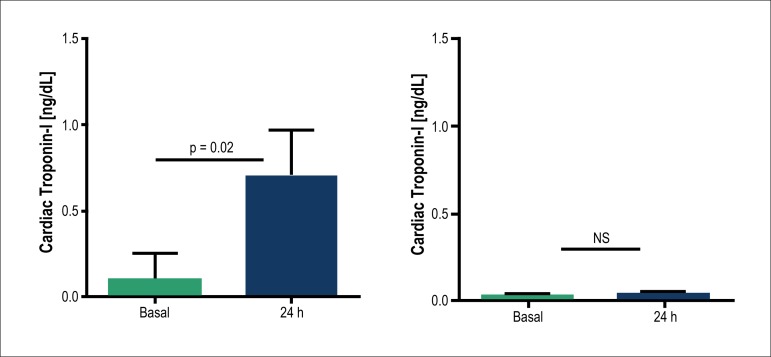
Changes in serum concentrations of troponin-I at baseline and 24 hours after percutaneous coronary intervention in control (left) and preconditioning (right) groups. Data are expressed as means ± SEM.

Changes in cTn I. Figure 2 display changes in serum concentrations of cTn I at baseline and 24 hours in both study groups. Patients in the control group presented a small but significant increase in cTn. Meanwhile, the marker remained unchanged in the preconditioned group.

**Figure 3:**
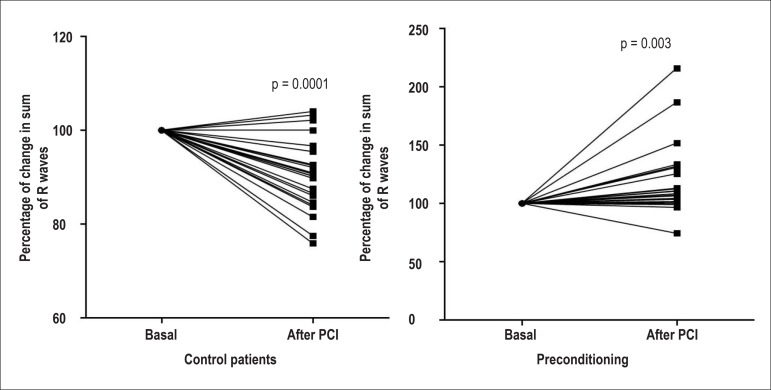
Percent changes in summation of R waves on all leads, from baseline to 24 hours after percutaneous coronary intervention (PCI) in control (left) and preconditioning (right) groups

**Figure 4:**
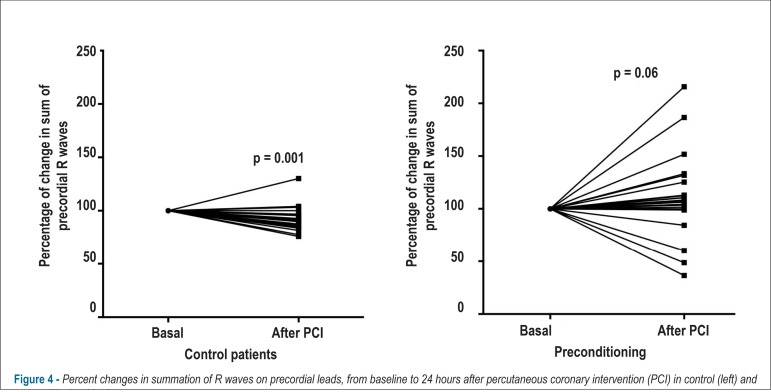
Percent changes in summation of R waves on precordial leads, from baseline to 24 hours after percutaneous coronary intervention (PCI) in control (left) and preconditioning (right) groups.

ECG changes. Four patients in the control and only one in the preconditioning groups had ST elevation greater than 1mV 24 hours after PCI (a non-significant statistical difference of 17.3% vs. 4.3%). Figure 3 shows the percentage of changes observed in the summation of R waves in all 12 ECG leads, while Figure 4 exhibits those changes just in precordial leads.

Table 2 displays the effect of the PCI procedure on R wave summation in control patients, as well as the effect of the preconditioning maneuver on that variable.

**Table 2:** R wave summation in control and experimental groups

**Total R summation (Σ)**					
**Control Group, X±SD, mm**			**Preconditioning group, X±SD, mm**		
**Σbasal**	**post PCI**	**p value**	** Σbasal**	**post PCI**	**p value**
56.9±139	51.3±12.7	< 0.0001	53.3±230	62.0±22.4	0.006
**Precordial R wave summation**					
**Σbasal**	**Σpost PCI**	**p value**	** Σbasal**	**Σpost PCI**	**p value**
36.5±21.8	32±17.9	0.003	33.1±18	37.3±19.5	0.057

SD: standard deviation; PCI: percutaneous coronary interventions.

As a whole, it can be seen that PCI procedure had a clear-cut effect on the summation of R waves, in all or only in precordial leads. In comparison, preconditioning maneuver not only preserved but also enhanced the summation of R waves. All these changes in the control and experimental groups reached statistical significance.

Renal data. Contrast media injected did not differ between groups (225.7 ± 10.27 and 221.3 ± 13.36 mL, for control and preconditioning groups, respectively, p= 0.79). Table 3 shows the effect of PCI on serum creatinine and GFR. In control group, creatinine rose 0.11 mg/dL (+8.6%), while in the preconditioning group, creatinine increased more, 0.17 mg/dL (+17%). Nevertheless, GFR descended 6 mL/min/1.73m^2^ (-9.5%) in control patients, whilst in preconditioning patients, it fell 6.7 (-10.6%). None of those differences were statistically significant.

**Table 3:** Serum creatinine and GFR values in control and experimental patients before and after PCI

**Control**			**Preconditioning**		
**Cr basal**	**Cr post PCI**	**p**	**Cr basal**	**Cr post PCI**	**p**
**mg/dL**	**mg/dL**	**value**	**mg/dL**	**mg/dL**	**value**
1.28±0.63	1.39±0.67	0.001	1.0±0.32	1.17±0.36	0.002
GFR mL/min/1.73 m^2^			GFR mL/min/1.73 m^2^		
**Basal**	**Post-PCI**		**Basal**	**Post-PCI**	
63±26.5	57±23.5	0.003	72.0±21.1	64.4±20.97	<0.0001

Cr: serum creatinine; GFR: glomerular filtration rate; PCI: percutaneous coronary intervention. *No intergroup significant differences were found.

## Discussion

The main findings of this work showed that a RIPC induced an intracoronary increase of NO levels resulting in a decrease in myocardial damage (measured as no increase in cTn-I) with electrocardiographic increases in the summation of R waves, suggesting an improved myocardium after elective PCI.

The mechanisms by which repeated episodes of ischemia-reperfusion in a non-cardiac tissue or organ lead to subsequent myocardial protection against ischemia are not completely understood. The effect of brief limb ischemia has been studied in both animals and humans.^5^ The work of Loukogeorgais et al.^14^ demonstrated that limb occlusion in one arm diminishes endothelial dysfunction in the contralateral arm. Several hypothetical mechanisms have been proposed to explain this phenomenon, mainly the neural and humoral hypothesis. Remote tissue subject to ischemia-reperfusion must produce one or several substances (adenosine, bradykinin, calcitonin gene-related peptide, endogenous opioids, among others) that can stimulate an efferent neural pathway with cardioprotective results or traversing the blood stream can act directly in the endothelium of the coronary vessels, inducing a preservation response (that is, reversing endothelial dysfunction).^6^ Other proposed mechanism is the activation of the enzymatic system of mitogen-activated protein kinases (MAPKs) p38, Erk1/2 and JNK, in the remote tissue subjected to ischemia-reperfusion that can exert positive modifications yielding to ischemic protection in the distant myocardium.^15^ More recently, the role of NO has emerged as the pivotal mechanism explaining ischemia protection, classical or remote. There is evidence about the induction by RIPC of increased activation of endothelial NO synthase (eNOS), rather than increased expression,^16^ as well as an increase of NO production and its oxidation products nitrites/nitrates. In elegant experiments using the rat cremaster flap *in vivo* microscopy model, Küntscher et al. have demonstrated that NO caused higher capillary flow and faster red blood cell velocity in arterioles and capillaries, while L-nitroarginine methylesther (L-NAME), a direct inhibitor of NOS, inhibits the preconditioning effect.^17^ NO, the signaling molecule iconic of endothelial function, exerts plentiful biological actions that can explain its cardiovascular protection effects: modulation of excitability, attenuation of cellular stress response, arteriolar and capillary dilation, antioxidant, antiinflammatory, antifibrotic, antithrombotic and antiapoptotic effects, among others. Besides, NO functions also as an intracellular messenger, a paracrine molecule, a neurotransmitter, or even as a hormone with different distant effects, generally beneficial.^18^

Although our work demonstrated a clear increase in NO (indirectly measured through its degradation products, NO_2_/NO_3_) levels in the vicinity of coronary atherosclerotic plaque after remote preconditioning, just before angioplasty intervention, the metabolic changes caused by the preconditioning maneuver are not at all the only source of NO production. It is now known, for example, that nitrite can be the source of NO mainly in ischemic or hypoxic conditions, besides the classical NO synthase pathway.^19^ Even though, the production of NO through the preconditioning phenomenon is still an explanation of the myocardium salvage shown in our study (comparatively lower levels of serum cTn, and preservation or gain of electrocardiographic R waves after the coronary intervention), as the rescue was seen in the preconditioned patients and not in the controls.

On this regard, the sensitivity of current necrosis biomarkers makes it possible to detect minute myocardial necrosis, making apparently easier the diagnosis of this clinical entity. However, the criteria for the diagnosis of myocardial infarction and periprocedural myocardial infarction have been modified several times in the last few years, introducing considerable confusion in the matter.^19^ According to current concepts, increases of necrosis markers above the 99^th^ percentile of the reference value are defined as myocardial necrosis ("myonecrosis"), while an increase of at least five times above the reference value of the 99^th^ percentile supports the diagnosis of myocardial infarction.^20^ This graded diagnostic differentiation is based on the fact that a small increase in biomarkers can be seen in multiple conditions, such as heart failure, myocarditis, myocardiopathy, renal insufficiency, pulmonary thromboembolism, fast or slow arrhythmias, ventricular hypertrophy, cardiac toxicity (v.gr., anthracyclines), heart surgery and trauma, anemia, shock and sepsis, among many more.

The importance of periprocedural myocardial infarction or periprocedural myonecrosis resides obviously in the amount of viable myocardial lost during coronary manipulation, and, for this reason, it seems commonsensical prevent or at least limit the occurrence of myocardial injury and its extension. Periprocedural myocardial infarction is less clinically relevant than spontaneous myocardial infarction, as it was shown in the ACUITY trial,^21^ in which the former was associated with a mortality relative risk of 7.49, whereas, in the latter, mortality did not increase.

Our study showed that in the control group a definite periprocedural myocardial loss occurred. In those patients, mean cTn value increased seven times (0.1 to 0.7 ng/dL) from baseline to post-intervention, meaning 17.5 times the 99^th^ percentile reference value of 0.04 ng/dL. While, at baseline, 11 patients had cTn values greater than 0.04, in post-PCI that number increased to 19. In comparison, in the experimental group, mean cTn values did not exceed the 99^th^ percentile reference value both at baseline and post-PCI (0.02 to 0.04 ng/dL). At baseline, 8 patients had cTn values greater than 0.04, and after PCI only 9 showed a high value. These results call attention to the salvage of myocardium during PCI with the use of an easy to do, cheap and almost harmless preconditioning maneuver.

Furthermore, the myocardial preservation effect of preconditioning was confirmed in our study with ECG data. ECG is a time-honored diagnostic tool in the clinical recognition of myocardial infarction, its topographic localization, estimation of infarction size and prognosis. Several indexes and scoring electrocardiographic systems were developed to reflect the functional state of the left ventricle in times previous to image technologies apt to estimate very accurately systolic and diastolic properties of the left ventricle. Unfortunately, the correlation among several ECG scores and ejection fraction was rather poor.^22^ More recently, in the era of coronary intervention, the meaning of the evolution of R wave's voltages in different phases of myocardial ischemia and reperfusion has attracted again more attention.^23^ In the classical electrophysiological explanation, electric activated myocardial cells generate action potentials represented by vectors, whose frontal heads are positively charged. Necrotic or extremely ischemic myocardial cells cannot generate these electrical forces. Diverse clinical and experimental studies have shown that R wave amplitude decreases notoriously during acute ischemia, but increases its voltage during reperfusion.^23,24^ This phenomenon probably indicates that during an undetermined period of time myocardial cells attacked by ischemia are not dead, but just in a critical *pre-mortem* state of extreme injury, keeping only the basic supporting life systems. But if flow is reestablished, these myocardial ischemic cells can restore fully its vitality and functionality. It has been described that, in humans, undergoing coronary angioplasty, during the brief episodes of ischemia caused by balloon inflation, R wave amplitude increases significantly in almost all ECG leads. Although the real cause of this voltage gain in the summation of R wave has not been convincingly disclosed (maybe the result of left ventricular cavity expansion or conduction alterations), anyhow this phenomenon is associated with acute ischemia, different from the phenomenon observed in this work.^25^ So, the amplitude variation of R waves represents this "entrance and exit" of the cells in the ischemic dimly zone. Our data show that, while in control patients myocardial loss occurred, in patients who underwent a preconditioning maneuver, myocardium was better preserved.

On the other hand, our results showed that renal function was impaired in both groups. The magnitude of the renal derangement was noticeable, but not prominent, far away from the boundaries of the contrast-induced nephropathy (a 25% increase of serum creatinine or 0.5 mg/dL absolute augment).^26^ In our patients, the preconditioning maneuver had no positive change in renal functionality.

## Conclusion

The easy to do, inexpensive and harmless arm cuff occlusion maneuver carried out before PCI can protect from myocardial damage caused by coronary intervention itself and post-angioplasty reperfusion lesion, due to greater NO bioavailability in the atherosclerotic lesion.

### Study limitations

Even with a clear participation of NO in decreasing myocardial damage, the complexity of preconditioning phenomena still needs more profound studies. For example, that phenomenon is known to have two phases, an early one ("first window"), beginning almost immediately and lasting several hours, and a delayed one ("second window"), starting 12 to 24 hours later and with a longer duration of 48-72 hours, termed the "Second Window of Protection" (SWOP) or late ischemic preconditioning.^6^ While in the first "window" myocardial preconditioning provokes vasodilation, in the second "window", various downregulation changes occurred in proteins and the expression genes encoding several proteins related to oxidation, heat shock protective response, and activation of NF-κB, among others. Several other investigations point out that NO can act as a mediator of "second-window" ischemic preconditioning^27^ over the opening of the end effector of late preconditioning, the mitochondrial ATP-sensitive potassium (mitoK-ATP)^28^ channel. In addition, it has to be proven that NO acts in both windows of the preconditioning phenomenon, as well as which is the isoenzyme responsible for its increase production (eNOS or iNOS).

Finally, the clinical and prognostic consequences of the myocardium salvage resulting from the preconditioning maneuver have yet to be tested in the long range.
